# Supplementary material for: Effect of Fetoscopic Laser Photocoagulation on Fetal Growth and Placental Perfusion in Twin–Twin Transfusion Syndrome
Source: J Clin Med. 2022 Jul 28;11(15):4404. doi: 10.3390/jcm11154404 (PMC9368961; doi:10.3390/jcm11154404)
Supplement: Supplementary file 1 [file jcm-11-04404-s001.zip › jcm-1755134-supplementary.pdf]

Table S1. Post hoc (LSD) tests for multiple comparisons of BWDs in M1,M2 and M3

LSD

Dependent Variable	(I) stage	(J) stage	Mean Difference (I-J)	Std. Error	Sig.	95% Confidence Interval	
						Lower Bound	Upper Bound
BWD in M1	1	2	4.91747	5.54995	.380	-6.2356	16.0705
		3	-9.59472	6.16842	.126	-21.9906	2.8012
		4	-8.22655	7.41352	.273	-23.1246	6.6715
	2	1	-4.91747	5.54995	.380	-16.0705	6.2356
		3	-14.51220*	4.66289	.003	-23.8826	-5.1418
		4	-13.14402*	6.21719	.040	-25.6379	-.6501
	3	1	9.59472	6.16842	.126	-2.8012	21.9906
		2	14.51220*	4.66289	.003	5.1418	23.8826
		4	1.36817	6.77502	.841	-12.2467	14.9831
	4	1	8.22655	7.41352	.273	-6.6715	23.1246
		2	13.14402*	6.21719	.040	.6501	25.6379
		3	-1.36817	6.77502	.841	-14.9831	12.2467
BWD in M2	1	2	1.02410	7.24705	.888	-13.5394	15.5876
		3	-14.98008	8.05465	.069	-31.1665	1.2063
		4	-17.97862	9.68048	.069	-37.4323	1.4750
	2	1	-1.02410	7.24705	.888	-15.5876	13.5394
		3	-16.00418*	6.08874	.011	-28.2400	-3.7684
		4	-19.00273*	8.11832	.023	-35.3171	-2.6884
	3	1	14.98008	8.05465	.069	-1.2063	31.1665
		2	16.00418*	6.08874	.011	3.7684	28.2400
		4	-2.99854	8.84673	.736	-20.7767	14.7796
	4	1	17.97862	9.68048	.069	-1.4750	37.4323
		2	19.00273*	8.11832	.023	2.6884	35.3171
		3	2.99854	8.84673	.736	-14.7796	20.7767
BWD in M3	1	2	.47303	4.72121	.921	-9.0146	9.9607
		3	-8.50417	5.24733	.112	-19.0491	2.0407
		4	-2.73749	6.30650	.666	-15.4109	9.9359
	2	1	-.47303	4.72121	.921	-9.9607	9.0146
		3	-8.97720*	3.96661	.028	-16.9484	-1.0060

	4	-3.21052	5.28881	.547	-13.8388	7.4177
3	1	8.50417	5.24733	.112	-2.0407	19.0491
	2	8.97720*	3.96661	.028	1.0060	16.9484
	4	5.76668	5.76335	.322	-5.8152	17.3486
4	1	2.73749	6.30650	.666	-9.9359	15.4109
	2	3.21052	5.28881	.547	-7.4177	13.8388
	3	-5.76668	5.76335	.322	-17.3486	5.8152

*. The mean difference is significant at the 0.05 level.
